# Development of a Hyperosmotic Stress Inducible Gene Expression System by Engineering the MtrA/MtrB-Dependent *NCgl1418* Promoter in *Corynebacterium glutamicum*

**DOI:** 10.3389/fmicb.2021.718511

**Published:** 2021-07-21

**Authors:** Jingwen Huang, Jiuzhou Chen, Yu Wang, Tuo Shi, Xiaomeng Ni, Wei Pu, Jiao Liu, Yingyu Zhou, Ningyun Cai, Shuangyan Han, Ping Zheng, Jibin Sun

**Affiliations:** ^1^School of Biology and Biological Engineering, South China University of Technology, Guangzhou, China; ^2^Key Laboratory of Systems Microbial Biotechnology, Tianjin Institute of Industrial Biotechnology, Chinese Academy of Sciences, Tianjin, China; ^3^College of Biotechnology, Tianjin University of Science and Technology, Tianjin, China

**Keywords:** *Corynebacterium glutamicum*, hyperosmotic stress, inducible promoter, MtrA, fluorescence activated cell sorting, lysine

## Abstract

*Corynebacterium glutamicum* is an important workhorse for industrial production of diversiform bioproducts. Precise regulation of gene expression is crucial for metabolic balance and enhancing production of target molecules. Auto-inducible promoters, which can be activated without expensive inducers, are ideal regulatory tools for industrial-scale application. However, few auto-inducible promoters have been identified and applied in *C. glutamicum*. Here, a hyperosmotic stress inducible gene expression system was developed and used for metabolic engineering of *C. glutamicum*. The promoter of *NCgl1418* (P*_NCgl1418_*) that was activated by the two-component signal transduction system MtrA/MtrB was found to exhibit a high inducibility under hyperosmotic stress conditions. A synthetic promoter library was then constructed by randomizing the flanking and space regions of P*_NCgl1418_*, and mutant promoters exhibiting high strength were isolated *via* fluorescence activated cell sorting (FACS)-based high-throughput screening. The hyperosmotic stress inducible gene expression system was applied to regulate the expression of *lysE* encoding a lysine exporter and repress four genes involved in lysine biosynthesis (*gltA*, *pck*, *pgi*, and *hom*) by CRISPR interference, which increased the lysine titer by 64.7% (from 17.0 to 28.0 g/L) in bioreactors. The hyperosmotic stress inducible gene expression system developed here is a simple and effective tool for gene auto-regulation in *C. glutamicum* and holds promise for metabolic engineering of *C. glutamicum* to produce valuable chemicals and fuels.

## Introduction

The nonpathogenic Gram-positive *Corynebacterium glutamicum* is a biosafe strain recognized by FDA and widely used in biomanufacturing of amino acids, organic acids, proteins, and other chemicals (Woo and Park, 2014; Lee et al., 2016; Tsuge and Matsuzawa, 2021). Regulation and optimization of target gene expression is crucial for balance of metabolic pathway and improvement of product biosynthesis. Promoters with different properties are one of the most effective tools to control gene expression. At present, a series of promoters have been identified or developed to regulate gene expression in *C. glutamicum* (Becker et al., 2005; Kind et al., 2010; Patek and Nesvera, 2011; Dostalova et al., 2017; Zhang et al., 2018; Sun et al., 2020; Wang et al., 2020; Wiechert et al., 2020a,b; Henke et al., 2021). In general, there are two types of promoters: constitutive promoters and inducible promoters. The strong constitutive promoters, such as the *tuf* and *sod* promoters, are widely used to enhance the expression of target genes (Becker et al., 2005; Kind et al., 2010). However, in some cases, gene expression controlled by strong constitutive promoters may cause cellular burden and metabolic unbalance, which hinders bioproduction (Giacalone et al., 2006; Terpe, 2006). Compared to constitutive promoters, inducible promoters can initiate the gene expression at any time when required, so they are preferred for the regulation and redistribution of metabolic flux and the expression of proteins with cytotoxicity (Lipscomb et al., 2004). At the moment, *tac*, *trc*, and other inducible promoters are extensive applied in the metabolic regulation of *C. glutamicum* (Ben Samoun et al., 1999; Okibe et al., 2010; Lausberg et al., 2012; Sun et al., 2020; Wiechert et al., 2020b; Henke et al., 2021), but there are also limitations. The requirement of inducers that are toxic [such as isopropyl-β-*d*-thiogalactopyranoside (IPTG)] or exhaustible (such as gluconate) limits the use in a large scale. Besides, inducers are usually added during the cultivation, so monitoring the cell growth state and optimizing the timing of inducer addition are required (Nocadello and Swennen, 2012). Therefore, there is a demand in development of auto-inducible systems without the use of external inducers.

Recently, auto-inducible promoters that can automatically turn on gene expression in response to environmental factors, metabolites, and cell growth state have been identified and applied in several bacteria. For example, a dynamic turn-off switch (dTFS) and a dynamic turn-on switch (dTNS) in *Escherichia coli* were constructed using the growth phase-dependent promoter and degron (Lemke et al., 2011). This bifunctional molecular switch was used to uncouple cell growth from the biosynthesis of shikimic acid and glucaric acid (Hou et al., 2020). Pyruvate-responsive genetic circuits were constructed in *Bacillus subtilis* by the hybrid promoter that contained the PdhR-binding site and the pyruvate-responsive transcription factor PdhR from *E. coli*, which were used to balance and optimize the metabolic flux toward the production of glucaric acid (Ogasawara et al., 2007; Xu et al., 2020a). An endogenous quorum-sensing (QS) based CRISPRi circuit in *Streptomyces* was constructed, in which the *dcas9* gene was regulated by a native QS signal-responsive promoter. The system was used for downregulating three key nodes in essential pathways to divert metabolic flux toward rapamycin biosynthesis (Tian et al., 2020). In *C. glutamicum*, some auto-inducible promoters have also been reported and applied. For example, based on the LysR-type transcriptional regulator (LTTR) LysG and the *lysE* promoter with the LysG-binding site, a sensor suitable for intracellular lysine detection was developed (Binder et al., 2012). Two growth-regulated promoters (P*_cg3141_* and P*_CP 2836_*) had been identified (Kim et al., 2016; Ma et al., 2018). The P*_cg3141_* promoter was verified with the production of glutathione S-transferase as a model protein, and the P*_CP_2836_* promoter was used for improving the production of valine and 5-aminolevulinic acid (Kim et al., 2016; Ma et al., 2018; Zhang et al., 2020).

With strict control of fermentation conditions such as pH and dissolved oxygen, the titer of target metabolites in the bioreactors can reach 100 g/L or even 200 g/L, Lys production by *C. glutamicum*, for example (Xu et al., 2020b). Therefore, in the mid- and late-stage of fermentation, the accumulation of high concentrations of products or the feeding of substrates will inevitably lead to a high-salt or hyperosmotic stress, which can be used as a natural inducer that exits in almost all fermentation processes (Varela et al., 2003; Chung et al., 2017). However, hyperosmotic stress inducible promoters have not been identified so far. MtrA/MtrB, one of the two-component systems that are highly conserved in corynebacteria and mycobacteria, regulates the expression of genes involved in osmoprotection (Moker et al., 2004; Bott and Brocker, 2012). Some target genes and the consensus binding sites of MtrA have also been identified in *C. glutamicum* (Brocker et al., 2011). However, the MtrA/MtrB-dependent promoters responding to the hyperosmotic stress have not been identified and applied in gene regulation.

In this study, a hyperosmotic stress inducible gene expression system based on the hyperosmotic stress inducible promoter was developed in *C. glutamicum*. Among eight promoters regulated by the two-component system MtrA/MtrB, the promoter of *NCgl1418* (P*_NCgl1418_*) showed high expression intensity and induction activity under a hyperosmotic stress. The core sequence of P*_NCgl1418_* was identified and the activity and inducibility was further enhanced by mutagenesis and fluorescence activated cell sorting (FACS)-based high-throughput screening. To demonstrate an application of this inducible system in metabolic engineering of *C. glutamicum*, it was used for overexpression of lysine exporter and CRISPR-dCpf1-mediated multiplex gene repression of four endogenous genes (Li et al., 2020) to maximize lysine production. This MtrA/MtrB-dependent hyperosmotic stress inducible system may also be useful for gene regulation in microorganisms beyond *C. glutamicum*.

## Materials and Methods

### Bacterial Strains and Cultivation Conditions

The bacterial strains used in this study are listed in Supplementary Table S1. *E. coli* Trans1-T1 and Trans DB 3.1 were used as the host for cloning and plasmid maintenance. Wild-type *C. glutamicum* ATCC 13032 was used as the host for gene expression and promoter screening. *C. glutamicum* SCgL30, the derivative of *C. glutamicum* ATCC 13032 with a feedback deregulated aspartokinase (T311I; Becker and Wittmann, 2012), was used for lysine production. *E. coli* was cultivated at 37°C in Luria-Bertani (LB) medium (5 g/L yeast extract, 10 g/L tryptone, and 10 g/L NaCl). Kanamycin (50 μg/ml) or chloramphenicol (20 μg/ml) was added to LB medium as required. *C. glutamicum* was cultivated at 30°C in BHI medium containing 37 g/L bovine brain heart extract, 10 g/L (NH_4_)_2_SO_4_, 0.2 g/L K_2_HPO_4_, 0.3 g/L NaH_2_PO_4_, and 0.5 g/L MgSO_4_·7H_2_O. For fluorescence intensity determination, *C. glutamicum* was cultivated in a defined medium (CGXIIY medium) containing 50 g/L glucose, 2 g/L yeast extract, 16.5 g/L NH_4_Cl, 5 g/L urea, 1 g/L KH_2_PO_4_, 1 g/L K_2_HPO_4_, 42 g/L MOPS, 0.25 g/L MgSO_4_, 0.01 g/L FeSO_4_·2H_2_O, 0.01 g/L MnSO_4_·H_2_O, 0.001 g/L ZnSO_4_·7H_2_O, 0.2 mg/L CuSO_4_, 0.02 mg/L NiCl·6H_2_O, 0.01 g/L CaCl_2_, 0.03 g/L protocatechuic acid, 0.2 mg/L biotin, and 0.1 mg/L vitamin B1. Kanamycin (25 μg/ml), chloramphenicol (5 μg/ml) or IPTG (1 mM) was added when necessary.

### Plasmid Manipulation

The plasmids and primers used in this study are listed in Supplementary Tables S1 and S2, respectively. The promoter regions [about 300 bp upstream of the coding sequence (CDS)] of *abgT*, *csbD*, *betP*, *NCgl1418*, *NCgl1756*, *NCgl1838*, *NCgl2841*, and *proP* were amplified from the genomic DNA of *C. glutamicum* ATCC 13032 by PCR with eight primer pairs abgT-F/R, csbD-F/R, betP-F/R, 1418-F/R, 1756-F/R, 1838-F/R, 2841-F/R, and proP-F/R, respectively. *E. coli*-*C. glutamicum* shuttle expression vector pXM-*gfp* (Sun et al., 2019) was used to characterize the promoter strength. The backbone of pXM-*gfp* was amplified by PCR with the primer pair pGFP-F/R and self-cyclized to construct the control plasmid pXM-con-*gfp* harboring no promoter for *gfp*. The promoters P*_abgT_*, P*_csbD_*, P*_betP_*, P*_NCgl1418_*, P*_NCgl1756_*, P*_NCgl1838_*, P*_NCgl2841_*, and P*_proP_* were individually inserted to pXM-con-*gfp* for *gfp* expression. The *tuf* promoter (the 349 bp upstream of the *tuf* gene) was amplified from the genomic DNA of *C. glutamicum* ATCC 13032 by PCR with the primer pair tuf-F/R. The backbone of pXM-P*_NCgl1418_*-*gfp* was amplified by PCR with the primer pair pGFP-tuf-F/pGFP-R. Then these two PCR fragments were purified and ligated using the CloneExpress® MultiS One Step Cloning Kit (Vazyme Biotech, Nanjing, China) to generate the plasmid pXM-P*_tuf_*-*gfp*. The backbone of pXM-*gfp* was amplified by PCR with the primer pair tac-F/R and then self-cyclized to generate the plasmid pXM-P*_tac_*-*gfp*. To test truncated P*_NCgl1418_* promoters, the P*_NCgl1418_* in pXM-P*_NCgl1418_-gfp* was replaced with truncated promoters, P*_NCgl1418-203_*, P*_NCgl1418-145_*, and P*_NCgl1418-94_*, by PCR using the forward primer 1418-203-F, 1418-145-F, and 1418-94-F, respectively, with the reverse primer pGFP-R.

*E. coli*-*C. glutamicum* shuttle expression vector pEC-XK99E was used to express *lysE*. The P*_NCgl1418_* promoter and the *lysE* gene were amplified by PCR using the primer pairs 1418-E-F/R and lysE-F/R from the genomic DNA of *C. glutamicum* ATCC 13032, respectively. Then the backbone of pEC-XK99E was amplified with the primer pair pEC-F/R. These three PCR fragments were purified and ligated to generate the plasmid pEC-P*_NCgl1418_*-*lysE*. The P*_NCgl1418_* on pEC-P*_NCgl1418_-lysE* was replaced with the P*_NCgl1418_* variant (P*_NCgl1418-A10_*) by PCR using the primer pair A10-E-F/R, generating the plasmid pEC-P*_NCgl1418-A10_-lysE*.

A previously developed CRISPR-dCpf1 system (Li et al., 2020) was modified to repress the expression of target genes in response to the hyperosmotic stress. To construct an all-in-one CRISPRi tool plasmid, the backbone of pXM-07 expressing dCpf1 was divided into three parts, which were amplified with the primer pairs pXM-07-F1/R1, pXM-07-F2/R2, and pXM-07-F3/R3, respectively. A gRNA cassette consisting of a constitutive promoter (P*_11F_*), two direct repeats (DRs), a *ccdB* flanked by two *Bbs*I sites, and a terminator was amplified from pEC-02 (Li et al., 2020) with the primer pair ccdB-F/R. These four PCR fragments were purified and ligated to generate the plasmid pXM-*dCpf1*. Two oligonucleotides (RFP-F/R) were annealed and assembled into *Bbs*I-digested pXM-*dCpf1* backbone using a Golden Gate assembly method, resulting in the plasmid pXM-*dCpf1*-RFP. Similarly, array-F1/R1, array-F2/R2, array-F3/R3, and array-F4/R4 were annealed, respectively, and assembled into *Bbs*I-digested pXM-*dCpf1* backbone, resulting in the plasmid pXM-*dCpf1*-4crRNA (the crRNA array targeting *gltA*, *pgi*, *hom*, and *pck*). All crRNAs used in this study are listed in Supplementary Table S3. To construct a hyperosmotic stress inducible CRISPRi system, the P*_NCgl1418_* promoter was amplified from the genomic DNA of *C. glutamicum* ATCC 13032 by PCR using the primer pair 1418-D-F/R. The backbone of pXM-*dCpf1* or pXM-*dCpf1*-4crRNA was amplified with the primer pairs pXM-D-F1/R1 and pXM-D-F2/R2, respectively. Then these three PCR fragments were purified and ligated to generate the plasmids pXM-P*_NCgl1418_*-con and pXM-P*_NCgl1418_*-*dCpf1*-4crRNA, respectively. The P*_NCgl1418_* on pXM-P*_NCgl1418_*-con and pXM-P*_NCgl1418_*-*dCpf1*-4crRNA was replaced with the P*_NCgl1418-A10_* variant by PCR using the primer pairs A10-D-F1/R1 and A10-D-F2/R2, generating the plasmid pXM-P*_NCgl1418-A10_*-con and pXM-P*_NCgl1418-A10_-dCpf1*-4crRNA, respectively.

### Construction of the MtrA-Deleted Strain

Deletion of the *mtrA* gene in *C. glutamicum* ATCC 13032 chromosome was performed by the CRISPR-Cas9 system (Liu et al., 2017). Plasmid pgRNA-ΔmtrA was construct by inserting the flanking regions of *mtrA* into pgRNA2 and replacing the base-pairing region of gRNA targeting *ldhA* with a base-pairing region targeting *mtrA* (N20, 5'-GTGGTGCTCGGTTTGGAATC-3'). A part of pgRNA2 backbone was amplified by PCR using primer pair gRNA-mtrA/pgRNA-1. N20 for *mtrA* was added to the backbone by primer gRNA-mtrA. A second part of pgRNA2 backbone was amplified by PCR using primer pair pgRNA-2/pgRNA-3. The 979 and 1,008 bp flanking regions of *mtrA* were amplified from the genomic DNA of *C. glutamicum* ATCC 13032 using the primer pairs mtrA-del-up-F/R and mtrA-del-down-F/R, respectively. The four fragments were ligated using the CloneExpress® MultiS One Step Cloning Kit (Vazyme Biotech, Nanjing, China) to yield pgRNA-ΔmtrA. The two plasmids (pgRNA-ΔmtrA and pCas9) were transformed into *C. glutamicum* ATCC 13032 by electroporation, and the screening of *ΔmtrA* mutants was performed in BHI plate with 0.05 mM IPTG. Gene deletion was verified by checking the sizes of the colony PCR fragments. Tool plasmids were cured from the edited mutant before it was used for subsequent experiments.

### Construction of Synthetic Promoter Libraries

The synthetic promoter libraries of P*_NCgl1418_* were constructed using the primer pairs P-Library-F1/R1 and P-Library-F2/R2 containing degenerated oligonucleotides (Ns) by PCR using pXM-P*_NCgl1418_-gfp* as a template. The PCR product was self-ligated with T4 ligase. The ligation products were transformed into strain *E. coli* Trans1-T1. The quality of randomization was checked by sequencing 24 randomly picked transformants. The synthetic promoter libraries were extracted and then transformed into *C. glutamicum* ATCC 13032 by electroporation.

### FACS Screening

The *C. glutamicum* library was inoculated into BHI medium and cultivated overnight at 30°C and 220 rpm. The overnight cultures were transferred into 24-well plates with 1 ml CGXIIY medium containing 0.6 M Na_2_SO_4_ to an optical density at 600 nm (OD_600_) of 0.5. Then, cells were cultivated for 6 h at 30°C and with shaking at 800 rpm in INFORS Microtron (INFORS HT Multitron Pro, Switzerland). The cell suspension was diluted by proper times with distilled water and sorted on FACS (MoFlo XDP, Beckman Coulter, Inc., Miami, FL, United States) using green fluorescent protein (GFP) as the reporter protein. The cells were excited by 488-nm laser and detected through a 520 band-pass filter. In the first-round screening, cells showing high fluorescence intensity (the top 0.01%) were sorted (positive sorting). The sorted cells were poured directly into 5 ml BHI medium and cultivated overnight, and then the cells were transferred into 24-well plates with 1 ml CGXIIY medium for the next round of screening. In the second-round screening, cells showing low fluorescence intensity (the bottom 1%) were sorted (negative sorting). Similarly, the third-round screening (positive sorting) was performed. After the three-round sorting, the sorted cells were spread on BHI agar plate and incubated at 30°C.

### Fluorescence Intensity Determination

The *C. glutamicum* was inoculated into BHI medium and cultivated overnight at 30°C and 220 rpm. The overnight cultures were transferred into 24-well plates with 1 ml CGXIIY medium with or without 0.6 M Na_2_SO_4_ to an OD_600_ of 0.5. After cultivated at 30°C and with shaking at 800 rpm for 18 h, cells were harvested and diluted by proper times with distilled water. GFP fluorescence intensities were determined using a microplate reader (SpectraMax M5, Molecular Devices, *λ* excitation = 488 nm, *λ* emission = 520 nm). The fluorescence intensities were normalized with OD_600_.

### Lysine Production in 24-Well Plates

*C. glutamicum* SCgL30 with a feedback deregulated aspartokinase (T311I; Becker and Wittmann, 2012) was used for lysine production. Strain SCgL30 and its derivatives were cultivated in BHI medium at 30°C and with shaking at 220 rpm. The overnight cultures were transferred into 24-well plates containing 1 ml fermentation medium each well to an initial OD_600_ of 0.5. The fermentation medium contains 80 g/L glucose, 8 g/L yeast extract, 9 g/L urea, 1.5 g/L K_2_HPO_4_·3H_2_O, 0.01 g/L MnSO_4_, 0.6 g/L MgSO_4_·7H_2_O, 0.01 g/L FeSO_4_·7H_2_O, and 42 g/L MOPS (Li et al., 2020). Then, cells were cultivated for 24 or 36 h at 30°C and with shaking at 800 rpm in INFORS Microtron (INFORS HT Multitron Pro, Switzerland).

### Lysine Production in 1-L Bioreactors

Colonies grown on a BHI plate at 30°C for 24 h were picked and used to inoculate 50 ml LSS1 medium (Ohnishi et al., 2002) in 500 ml shake flasks. The cultures were then cultivated overnight at 30°C and with shaking at 220 rpm. The cultures were transferred into a 1-L jar bioreactor [T&J-MiniBox, T&J Bio-engineering (Shanghai) Co., Ltd., China] containing 500 ml LPG1 medium (Ohnishi et al., 2002) with an inoculation volume of 10%. After the sugar initially added to the bioreactor was exhausted, glucose solution (50%, w/v) containing 0.5 mg/L biotin was continuously fed to maintain the glucose concentration at about 1.5% (NH_4_)_2_SO_4_ solution (5.56%, w/v) was also fed to supply nitrogen source for lysine biosynthesis and cell growth. The fermentation was performed at 34°C with an agitation speed of 900 rpm and an aeration at 2.5 L/min. The pH was maintained at 7.3 with NH_4_OH. LSS1 medium (Ohnishi et al., 2002) contains 50 g/L sucrose, 40 g/L corn steep liquor, 8.3 g/L (NH_4_)_2_SO_4_, 1 g/L urea, 2 g/L KH_2_PO_4_, 0.83 g/L MgSO_4_·7H_2_O, 10 mg/L FeSO_4_·7H_2_O, 1 mg/L CuSO_4_·5H_2_O, 10 mg/L ZnSO_4_·7H_2_O, 10 mg/L β-alanine, 5 mg/L vitamin B3, 1.5 mg/L vitamin B1, 0.5 mg/L biotin, and 30 g/L CaCO_3_. LPG1 medium (Ohnishi et al., 2002) contains 50 g/L glucose, 20 g/L corn steep liquor, 15 g/L (NH_4_)_2_SO_4_, 1 g/L urea, 2.5 g/L KH_2_PO_4_, 0.75 g/L MgSO_4_·7H_2_O, 50 mg/L FeSO_4_·7H_2_O, 13 mg/L MnSO_4_·5H_2_O, 50 mg/L CaCl_2_·2H_2_O, 6.3 mg/L CuSO_4_·5H_2_O, 1.3 mg of ZnSO_4_·7H_2_O, 5 mg/L NiCl_2_·6H_2_O, 1.3 mg/L CoCl_2_·6H_2_O, 1.3 mg/L (NH_4_)_6_Mo_7_O_24_·4H_2_O, 23 mg/L β-alanine, 14 mg/L vitamin B3, 7 mg/L vitamin B1, and 0.5 mg/L biotin. Samples were taken periodically and glucose and lysine concentrations were quantified using an SBA-40D biosensor analyzer (Institute of Biology of Shandong Province Academy of Sciences, Shandong, China). Cell biomass was determined as OD_600_ with a UV-1800 spectrophotometer (Shimadzu, Kyoto, Japan) after proper dilution with distilled water.

## Results

### Identification of Hyperosmotic Stress Inducible Promoters

**Figure 1:**
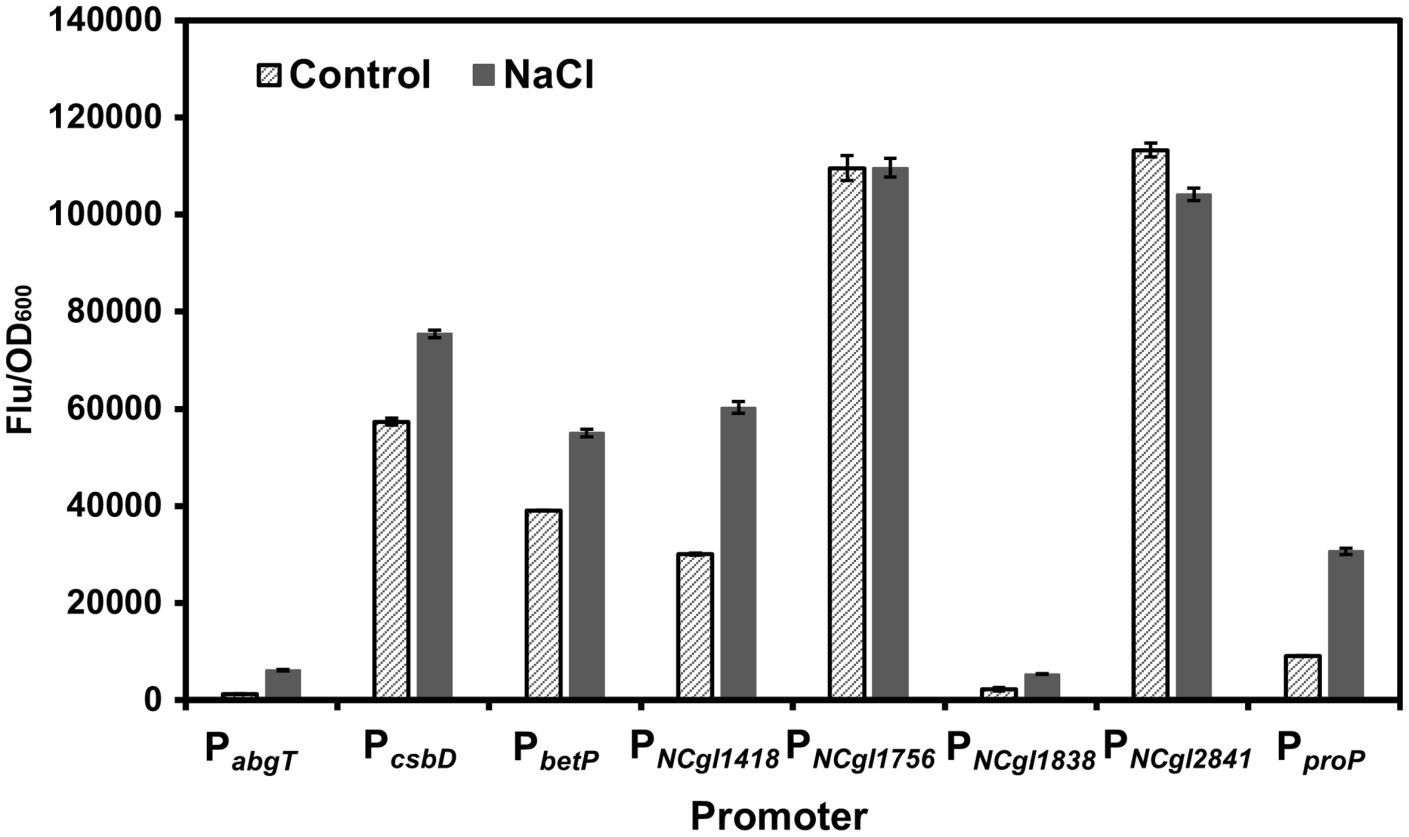
Screening of hyperosmotic stress inducible promoters. Expression of *gfp* was controlled by different MtrA/MtrB-dependent promoters (P*_abgT_*, P*_csbD_*, P*_betP_*, P*_NCgl1418_*, P*_NCgl1756_*, P*_NCgl1838_*, P*_NCgl2841_*, and P*_proP_*) in *C. glutamicum* with or without high salt stress induction. Cell fluorescence intensity was normalized to cell density and the background value of the strain containing the control vector pXM-con-*gfp* without a promoter was deducted. All data represent mean values from three biological replicates including standard deviations (SDs).

To identify the hyperosmotic stress inducible promoters, we tested the promoters of the genes showing MtrA-dependent behaviors. Eight promoters (P*_abgT_*, P*_csbD_*, P*_betP_*, P*_NCgl1418_*, P*_NCgl1756_*, P*_NCgl1838_*, P*_NCgl2841_*, and P*_proP_*), which control the expression of *abgT* (secondary transporter of the AbgT family), *csbD*, *betP* (glycine betaine transporter), *NCgl1418*, *NCgl1756*, *NCgl1838*, *NCgl2841*, and *proP* (proline/ectoine carrier), respectively, were selected based on a previous study (Brocker et al., 2011). These genes showed significantly decreased transcription levels (<10% of the control) upon the deletion of *mtrA* in *C. glutamicum*. Besides, the MtrA binding sites of these promoters have also been identified (Brocker et al., 2011). Since most of the exact promoters and regulatory regions of those genes have not been fully defined yet, the ~300 bp upstream of each CDS was investigated. In order to assess these MtrA-dependent promoters, GFP was used as a reporter to study the transcriptional strength of these promoters with or without 0.6 M NaCl. P*_abgT_*, P*_NCgl1418_*, P*_NCgl1838_*, and P*_proP_* showed more than 2.0-fold NaCl-induced expression activity. Among them, P*_NCgl1418_* showed the highest induced expression intensity (Figure 1). Therefore, we chose P*_NCgl1418_* for further analysis.

### Characterization of the *NCgl1418* Promoter

**Figure 2:**
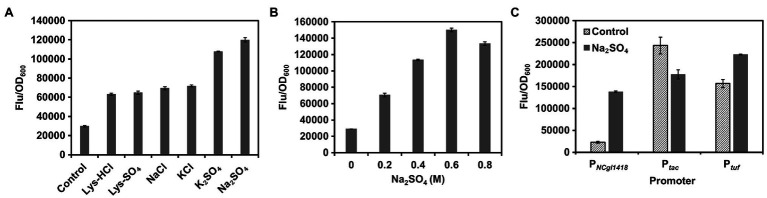
Characterization of P*_NCgl1418_* promoter. **(A)** Expression of *gfp* under the control of P*_NCgl1418_* in *C. glutamicum* induced by different salts. **(B)** Expression of *gfp* under the control of P*_NCgl1418_* in *C. glutamicum* induced by different concentrations of sodium sulfate. **(C)** Comparison of P*_NCgl1418_* with P*_tuf_* and P*_tac_* promoters. Expression of *gfp* was assayed with or without 0.6 M sodium sulfate. Cell fluorescence intensity was normalized to cell density and the background value of the strain containing the control vector pXM-con-*gfp* without a promoter was deducted. All data represent mean values from three biological replicates including SDs.

Next, the effects of different types or concentrations of inducers on induction of P*_NCgl1418_* were investigated. Using GFP as a reporter, it was found that sulfate was the best inducer, which induced the expression activity of P*_NCgl1418_* up to 4.0-fold (Figure 2A). P*_NCgl1418_* showed obvious gradient induction activity in the range of 0~0.6 M Na_2_SO_4_ (Figure 2B). Compared with the endogenous strong constitutive promoter P*_tuf_* and the widely used IPTG-inducible promoter P*_tac_* (Becker et al., 2005; Kind et al., 2010; Rytter et al., 2014), P*_NCgl1418_* showed about 62% expression strength upon hyperosmotic stress induction (Figure 2C). These results demonstrate that P*_NCgl1418_* is a relative strong hyperosmotic stress inducible promoter, which has application potential for initiating the expression of target genes during the fermentation process.

**Figure 3:**
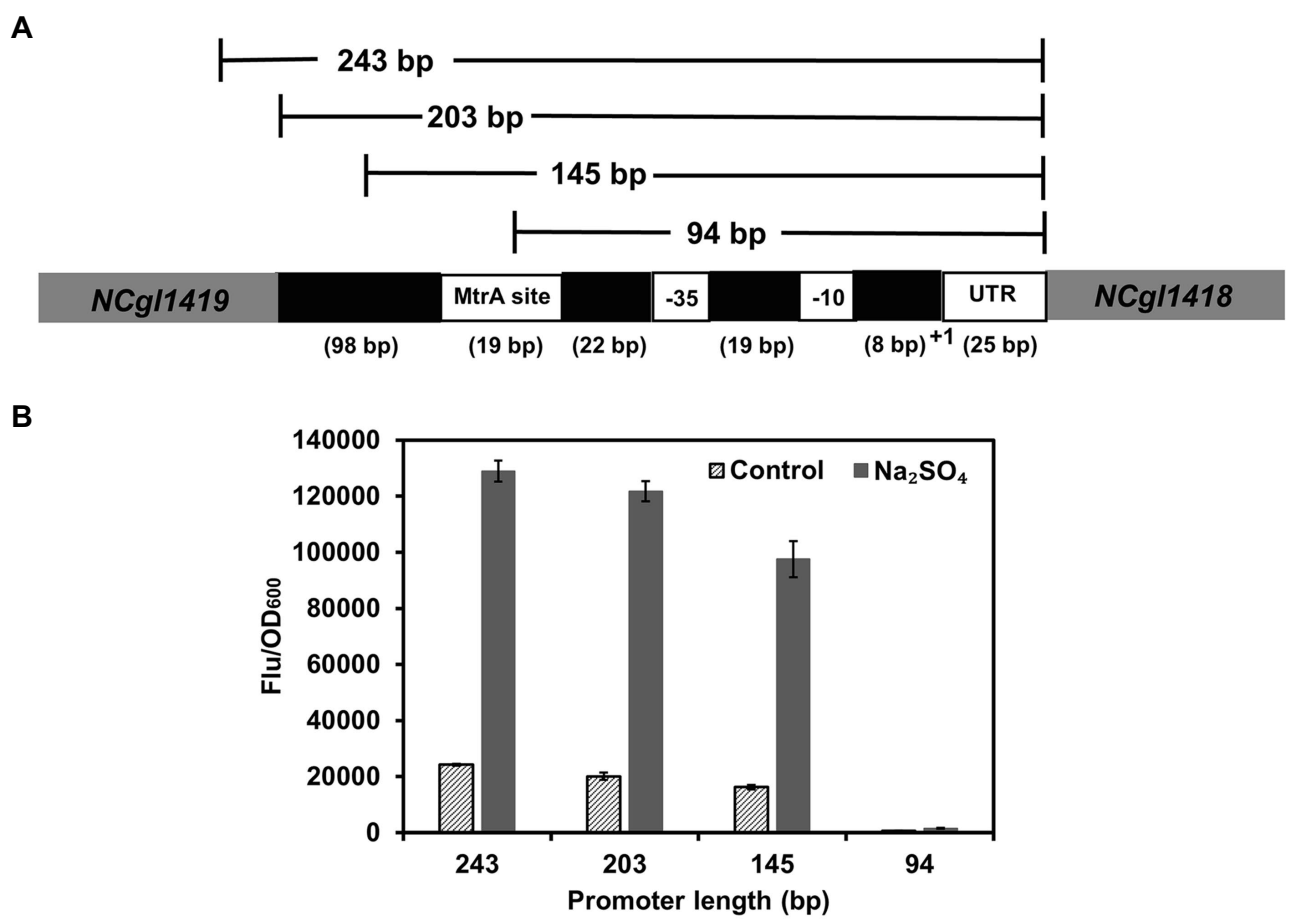
Identification of the core functional region of P*_NCgl1418_* promoter. **(A)** Schematic diagram of the P*_NCgl1418_* promoter truncation strategy. **(B)** Expression of *gfp* under the control of P*_NCgl1418_* promoter variants with different length (243, 203, 145, and 94 bp) with or without 0.6 M sodium sulfate. Cell fluorescence intensity was normalized to cell density and the background value of the strain containing the control vector pXM-con-*gfp* without a promoter was deducted. All data represent mean values from three biological replicates including SDs.

To determine the key functional region of P*_NCgl1418_*, three truncated variants (94, 145, and 203 bp) were constructed and used for GFP expression (Figure 3A). Although the 94 bp P*_NCgl1418_* promoter contained the core −35 and −10 regions (Pfeifer Sancar et al., 2013), the function of the promoter completely lost. The 145 bp promoter contained the MtrA binding site but showed a decreased expression activity (74%) under hyperosmotic stress. In contrast, the 203 bp promoter maintained almost the same activity (94%) to the whole-length promoter (243 bp) under hyperosmotic stress (Figure 3B). To confirm the necessity of MtrA for P*_NCgl1418_* functioning, a *mtrA*-deleted *C. glutamicum* strain was constructed and the plasmid harboring the P*_NCgl1418_*-controlled *gfp* cassette was transformed into the mutant. As expect, no GFP expression could be detected with *mtrA* deletion (Supplementary Figure S1). These results suggest that MtrA and its specific binding site are essential to the inducible activity of P*_NCgl1418_*. However, considering the partial activity loss of the 145 bp truncation, unknown mechanisms may also have an impact on the promoter.

### Construction of P*_NCgl1418_* Libraries and Isolation of Variants With Enhanced Inducible Activity

**Figure 4:**
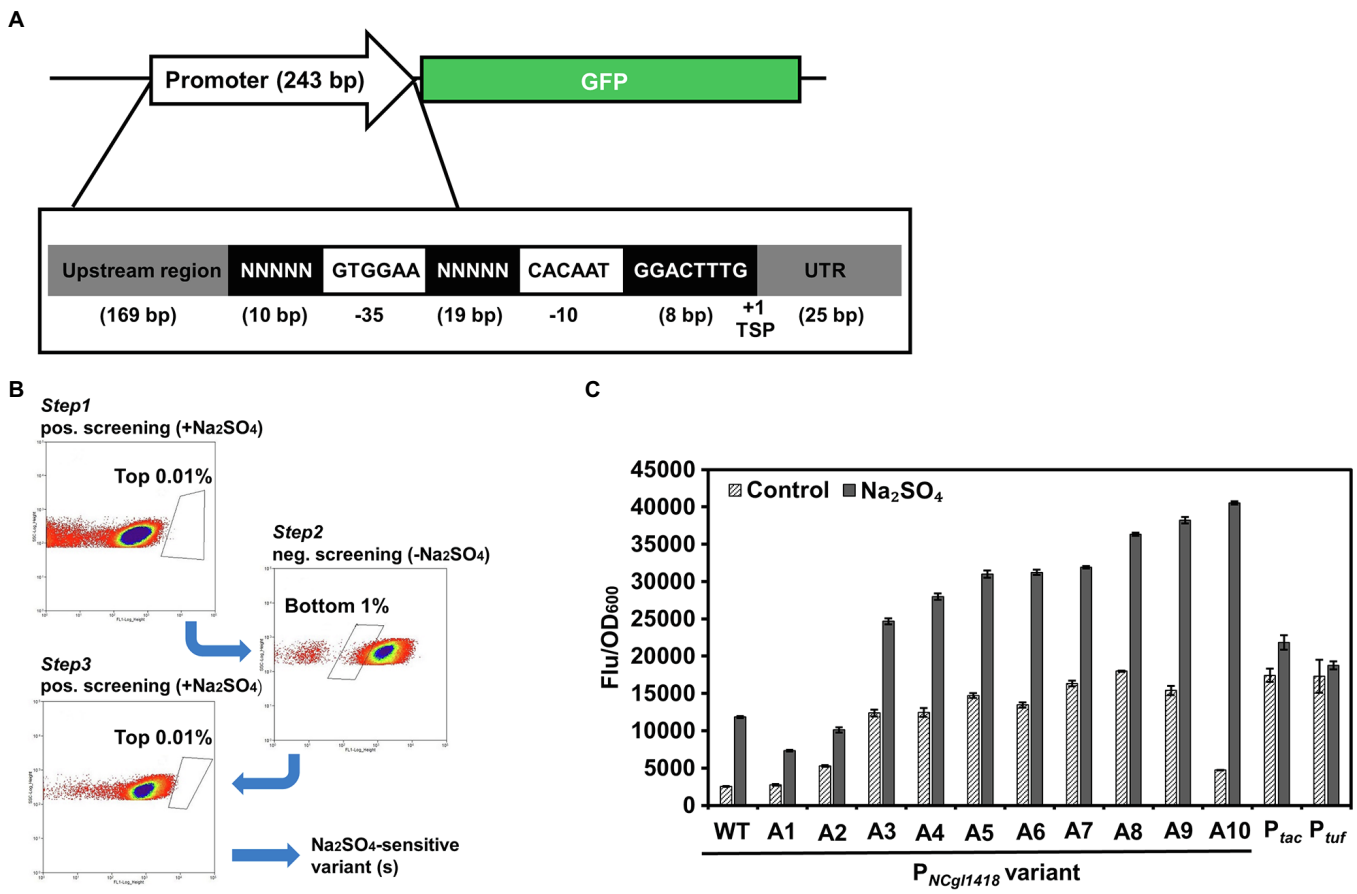
FACS-based positive/negative screening of the random promoter library to identify P*_NCgl1418_* promoter variants with enhanced inducible activity. **(A)** Design of the random promoter library. **(B)** FACS plots of the three rounds of positive/negative screening. **(C)** Characterization of the isolated promoter variants. Cell fluorescence intensity was normalized to cell density and the background value of the strain containing the control vector pXM-con-*gfp* without a promoter was deducted. All data represent mean values from three biological replicates including SDs.

Although P*_NCgl1418_* exhibited a relatively high inducible activity, its expression intensity was still lower than that of P*_tuf_* and P*_tac_*, which may hinder the application in high-level gene expression. Therefore, further engineering of this promoter is needed to increase its strength. To engineer the P*_NCgl1418_*, random promoter libraries were constructed based on the 243 bp wild-type promoter. The deduced −10 and −35 regions of P*_NCgl1418_* were fixed to allow sigma factor binding, and the 29 bp spacer and left flanking regions were designed to have fully randomized nucleotides (Figure 4A). The randomized promoters were cloned to the upstream of the GFP reporter gene and subjected to a three-step positive-negative-positive screening to identify promoter variants with improved induction activity and expression strength. During this screening process, positive screening in the presence of 0.6 M Na_2_SO_4_ was conducted to identify high-fluorescence variants (isolation of the top 0.01% fluorescing cells) and the negative screening in the absence of 0.6 M Na_2_SO_4_ was conducted to exclude the variants with too high fluorescence intensity without induction (isolation of the bottom 1% non-fluorescing cells; Figure 4B). After three rounds of screening, the sorted cells were cultivated on agar plates, and 90 clones were randomly picked and characterized. After a secondary screening in microtiter plates, a total of 10 promoter variants with different sequences (Supplementary Figure S2) and inducible activities were obtained (Figure 4C). One promoter variant A10 (P*_NCgl1418-A10_*) showed the highest inducible activity and was chosen for further analysis. P*_NCgl1418-A10_* was then compared with the strong constitutive promoter P*_tuf_* and IPTG-inducible promoter P*_tac_* with or without 0.6 M Na_2_SO_4_. The expression of GFP under the control of P*_NCgl1418-A10_* showed an 8.5-fold induction by 0.6 M Na_2_SO_4_. The induced GFP expression level was approximately 1.8-fold higher than those of P*_tuf_* and P*_tac_* (Figure 4C).

### Application of the Hyperosmotic Stress Inducible Promoter to Optimize Lysine Biosynthesis

**Figure 5:**
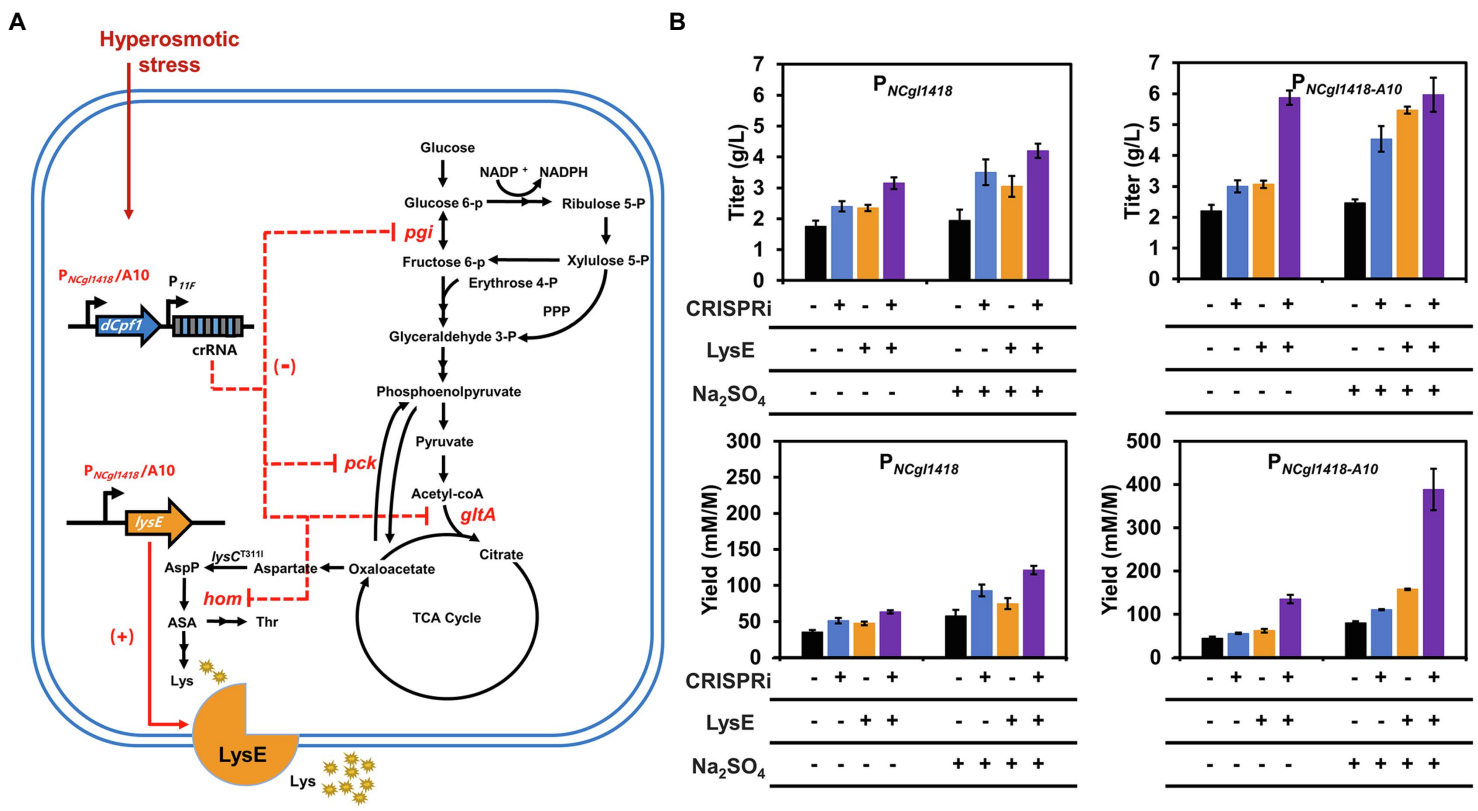
Enhancing lysine production by regulating the expression of target genes using the hyperosmotic stress inducible promoters. **(A)** Lysine biosynthesis pathway of *C. glutamicum*. (+) Represents gene overexpression and (−) represents gene repression. AspP, aspartyl phosphate; ASA, aspartate semialdehyde; Thr, threonine; Lys, lysine. **(B)** Effects of P*_NCgl1418_* and P*_NCgl1418-A10_* promoter-regulated *lysE* overexpression and/or multiple genes (*gltA*, *pgi*, *hom*, and *pck*) repression on lysine production. SCgL30 strains expressing LysE and/or dCpf1 with a crRNA array were cultivated in fermentation medium for 24 h (for P*_NCgl1418_* promoter) or 36 h (for P*_NCgl1418-A10_* promoter). SCgL30 strains containing the empty vector pEC-XK99E and/or the plasmid pXM-P*_NCgl1418_*-con without the crRNA array were used as controls. All data represent mean values from three biological replicates including SDs.

To demonstrate the usability of the hyperosmotic stress inducible promoters in metabolic engineering, the wild-type P*_NCgl1418_* and P*_NCgl1418-A10_* variant were used to regulate five genes related to lysine biosynthesis and excretion in *C. glutamicum* (Figure 5A). One target gene *lysE* encodes a lysine efflux protein and overexpression of *lysE* can increase lysine production by enhancing excretion of lysine (Zhou and Zeng, 2015). The other four genes, *gltA*, *pck*, *pgi*, and *hom*, are involved in the metabolic flux competition with lysine biosynthesis and their repression by CRISPRi has been proven to enhance the lysine production (Li et al., 2020).

Firstly, we constructed an all-in-one CRIPSRi tool plasmid pXM-*dCpf1* based on the previously developed two plasmids system expressing dCpf1 under the control of IPTG-inducible P*_tac_* and crRNA array under the control of constitutive P*_11F_* (Li et al., 2020). To determine the gene repression efficiency of the all-in-one CRIPSRi system, a crRNA targeting the template strand of red fluorescent protein (RFP) encoding gene was designed and assembled to the plasmid pXM-*dCpf1*. A repression efficiency of 77% was achieved (Supplementary Figure S3), which was similar with the previous system using two plasmids. For combinational repression of the four target genes (*gltA*, *pck*, *pgi*, and *hom*), a crRNA array harboring four spacers was assembled to pXM-*dCpf1*. To achieve hyperosmotic stress inducible gene repression, the IPTG-inducible P*_tac_* was replaced with P*_NCgl1418_* and P*_NCgl1418-A10_*, respectively, for controlling dCpf1 expression. The two CRISPRi plasmids and a control plasmid without targeting crRNA array were individually transformed into the lysine-producing strain SCgL30 with a feedback deregulated aspartokinase (T311I; Becker and Wittmann, 2012). Fermentation in 24-well plates was performed to evaluate lysine production. Without addition of 0.6 M Na_2_SO_4_ to provide a hyperosmotic stress, repression of four target genes using P*_NCgl1418_* enhanced lysine titer and yield by 37 and 45%, respectively (Figure 5B). When the hyperosmotic stress was exerted to cells by adding 0.6 M Na_2_SO_4_, repression of four target genes enhanced lysine titer and yield by 79 and 62%, respectively (Figure 5B). The lysine titer was improved by 92% (reached 4.5 g/L) when the stronger promoter variant P*_NCgl1418-A10_* was used in the presence of hyperosmotic stress. The results suggest that the hyperosmotic stress inducible promoters can be used for efficiently regulating gene expression under high salt conditions for metabolic engineering purposes.

For further testing the application of the hyperosmotic stress inducible promoters in gene upregulation, *lysE* was selected and overexpressed in SCgL30. Without the addition of 0.6 M Na_2_SO_4_, overexpression of *lysE* slightly increased the lysine titer and yield. However, under the hyperosmotic stress, *lysE* overexpression controlled by P*_NCgl1418-A10_* exhibited much higher lysine titer (2.3-fold) and yield (about 2.0-fold) compared with the control strain (Figure 5B). Finally, overexpression of *lysE* and repression of *gltA*, *pck*, *pgi*, and *hom* by CRISPRi were simultaneously performed for lysine production using the strain SLDEA10. Under the hyperosmotic stress, this combinational strategy improved lysine titer and the yield by 2.5- and 4.8-fold compared to the control, respectively (Figure 5B). It was noticed that the lysine titers of strain SLDEA10 cultivated with or without 0.6 M Na_2_SO_4_ were almost the same. This is may be due to the slower growth rate of strain SLDEA10 under hyperosmotic stress (Supplementary Figure S4). The growth retardation might be caused by the higher expression of dCpf1 and LysE or the stronger repression of the endogenous genes under hyperosmotic stress. However, the lysine yield reached 388.7 mM/M, which improved by 4.8-fold when 0.6 M Na_2_SO_4_ was added (Figure 5B). The results indicate that the promoter variant P*_NCgl1418-A10_* has strong inductivity and the rebalance between growth and production is necessary to increase the performance of engineered strains.

### Feed Batch Fermentation of the Lysine Producer With the Auto-Inducible System

**Figure 6:**
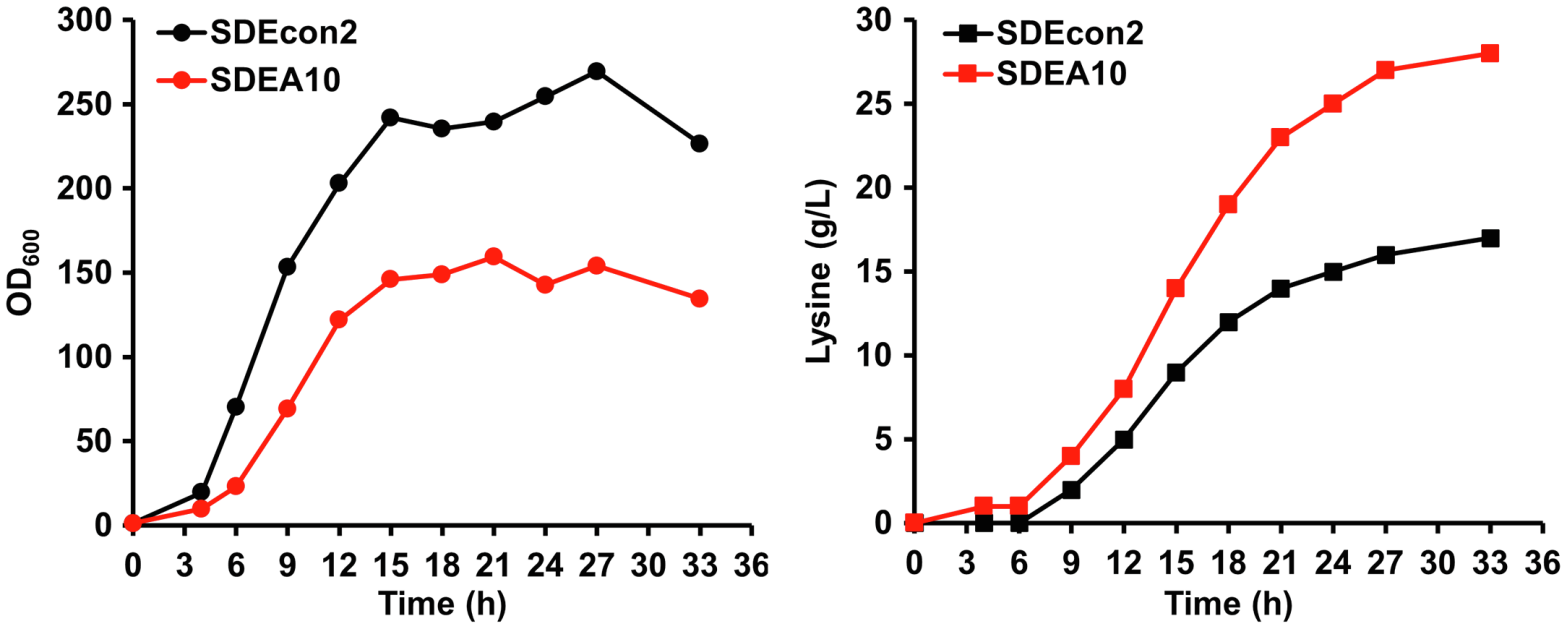
Fed-batch fermentation for lysine production using strains SDEcon2 (black lines) and SDEA10 (red lines). Strain SDEcon2 is strain SCgL30 harboring the empty vector pEC-XK99E and the plasmid pXM-P*_NCgl1418_*-con without the crRNA array. Strain SDEA10 is strain SCgL30 with overexpression of *lysE* and repression of *gltA*, *pgi*, *hom*, and *pck* controlled by P*_NCgl1418-A10_* promoter. Cultivation was performed in 1-L bioreactors with fermentation medium.

Because of the limited substrate supplement and the lack of fermentation condition control, fermentation in 24-well plates cannot result in high-titer lysine production. Therefore, 0.6 M Na_2_SO_4_ was added to provide a hyperosmotic stress to induce gene expression controlled by P*_NCgl1418_* and P*_NCgl1418-A10_*. Fed-batch fermentations are usually used to obtain high concentrations of products, which can serve as inducers for hyperosmotic stress inducible promoters such as P*_NCgl1418-A10_*. To demonstrate the auto-inducible system based on P*_NCgl1418-A10_*, fed-batch fermentations without exogenous Na_2_SO_4_ addition were conducted in 1-L bioreactors using the best lysine producer strain SLDEA10 and a control strain SLDEcon2 without the auto-inducible CRISPRi and *lysE* overexpression system. Overall, SLDEA10 had a better balance between growth and production than the control strain (Figure 6). Strain SLDEcon2 entered the stationary phage at 15 h with a relatively high OD_600nm_ peak value, while the highest OD_600nm_ was significantly reduced by 32.3% for strain SLDEA10 (Figure 6). On the contrary, the lysine biosynthesis rate of SLDEA10 was significantly higher than that of strain SLDEcon2 in the mid- and late-stage. After 33 h fermentation, 28.0 g/L of lysine was produced by strain SLDEA10, representing a 64.7% improvement compared to strain SLDEcon2 (17.0 g/L; Figure 6). These results suggest that the metabolic flux is directed from biomass synthesis to product synthesis by using the auto-inducible system based on the hyperosmotic stress inducible promoter.

## Discussion

In the last few decades, *C. glutamicum* has been engineered for the production of a variety of value-added products, including amino acids, organic acids, polymer precursors, and proteins (Baritugo et al., 2018; Lee and Kim, 2018). Gene expression regulation technologies based on CRISPR and synthetic small RNAs for *C. glutamicum* have been developed (Liu et al., 2017; Wang et al., 2018; Gauttam et al., 2019; Sun et al., 2019; Li et al., 2020). By combining these technologies with metabolic- or QS-responding elements, dynamic regulation strategies have been developed for precise regulation of gene expression to maximize the biosynthesis of target products (Gupta et al., 2017; Chen et al., 2018; Shen et al., 2019; Liang et al., 2020; Tian et al., 2020; Hartline et al., 2021). However, the metabolic-responding elements are specific for certain metabolites and thus they are not universal for various fermentation processes. Although QS systems have good universality, their application in large-scale fermentation usually suffers from the low threshold. As an alternative, the environmental parameter-responding elements have the advantages of high universality and good controllability, which can be used to develop universal dynamic regulation systems. At present, dynamic regulation strategies based on external environmental conditions such as temperature, dissolved oxygen, pH, and illumination have been successfully developed and applied in metabolic engineering (Jong Uk et al., 2008; Binder et al., 2016; Hwang et al., 2017; Harder et al., 2018; Zhao et al., 2018; Banares et al., 2020). However, in large-scale fermentation, these environmental factors including temperature, dissolved oxygen, and pH mostly need to be controlled at a steady level to maintain cellular activity, and optogenetics tools are limited by the nonhomogeneity in large fermenters. Hyperosmotic stress, which commonly presents in the mid- and late-phase of almost all the fermentation processes, can serve as a naturally occurring inducer. Compared with the promoters based on metabolic- or QS-responding elements and those responding to external environmental conditions such as temperature, dissolved oxygen, pH, and illumination, the hyperosmotic stress inducible promoters have good universality and economy in the large-scale industrialization applications. In this study, a hyperosmotic stress inducible gene expression system was developed in *C. glutamicum*. The promoter of *NCgl1418* identified here exhibits high expression intensity and induction activity under hyperosmotic stress caused by different salt inducers. More importantly, the induction activity was closely related to product concentration, which can be conveniently used to regulate the expression of key genes according to the product concentration. Considering the different expression intensities and induction activities by different types or concentrations of salts, optimization tests for different media may be required in practical applications.

Promoters are the core elements of the dynamic regulation systems and synthetic biology researches. Identification and modification of novel promoters with special functions can provide necessary tools for the development of new gene circuits and hyperproducing strains. At present, omics analysis under different culture conditions and bioinformatics analysis of specific recognition sequences of transcriptional regulators are the main strategies for the identification of new promoters (Dahl et al., 2013; Liu et al., 2015; Kim et al., 2016; Ma et al., 2018). However, the strength and induction activity of natural promoters are often not sufficient for practical applications. To engineer more suitable promoters, random mutation followed by high-throughput screening and redesign of the natural promoters with components from other different promoters are usually performed (Kim et al., 2016; Zhang et al., 2018; Henke et al., 2021). In this study, for isolation of engineered promoters with enhanced inducibility by hyperosmotic stress, we used a FACS screening strategy that combined three rounds of positive and negative screening. The isolated promoters showed up to a 3.4-fold increased GFP expression level compared to the original *NCgl1418* promoter with much higher inducibility (up to 8.5-fold). As a proof-of-concept, lysine production was improved by regulating key genes expression with the hyperosmotic stress inducible promoters, demonstrating the great potential in metabolic engineering of *C. glutamicum*. Also, we observed leaky expression of the *NCgl1418* promoter, like all the other kinds of inducible promoters. For a stricter control of the *NCgl1418* promoter, conditional overexpression of its regulator MtrA/MtrB may be a useful strategy. In addition, promoter variants with low-level leaky expression could also be further screened by FACS. In conclusion, we identified the MtrA/MtrB-dependent *NCgl1418* promoter as a hyperosmotic stress inducible promoter and developed a hyperosmotic stress inducible gene expression system in *C. glutamicum*. To the best of our knowledge, this is the first report on the development of a hyperosmotic stress inducible promoter in *C. glutamicum*. We believe this hyperosmotic stress inducible gene expression system will contribute to extend the usefulness of *C. glutamicum* in the industrial-scale production of various value-added biochemicals.

## Data Availability Statement

The original contributions presented in the study are included in the article/Supplementary Material, further inquiries can be directed to the corresponding authors.

## Author Contributions

JH, JC, YW, PZ, and JS conceptualized the project, designed the study, and composed the manuscript. JH, JC, TS, YZ, and NC conducted the experiments and collected data. JH, JC, YW, XN, WP, and JL performed the data analysis. PZ, JS, and SH provided the critical feedback on the manuscript and resources. All authors contributed to the article and approved the submitted version.

### Conflict of Interest

The authors declare that the research was conducted in the absence of any commercial or financial relationships that could be construed as a potential conflict of interest.
