# Supplementary material for: Development of a Hyperosmotic Stress Inducible Gene Expression System by Engineering the MtrA/MtrB-Dependent NCgl1418 Promoter in Corynebacterium glutamicum
Source: Front Microbiol. 2021 Jul 21;12:718511. doi: 10.3389/fmicb.2021.718511 (PMC8334368; doi:10.3389/fmicb.2021.718511)
Supplement: Supplementary file 1 [file Data_Sheet_1.PDF]

Supplementary Material

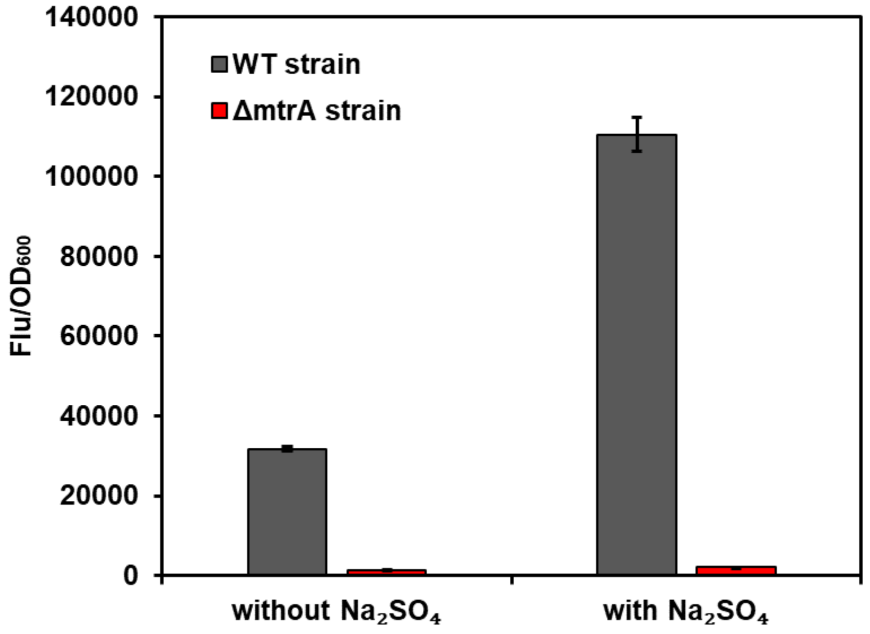

Figure S1. The expression of *gfp* gene under the $P_{NCgl1418}$ in wild-type *C. glutamicum* and *mtrA*-deficient (*ΔmtrA*) mutant. Cell fluorescence intensity was normalized to cell density and the background value of the strain containing the control vector pXM-con-*gfp* without a promoter was deducted. *C. glutamicum* ATCC 13032 containing the vector pXM-$P_{NCgl1418}$-*gfp* was used as control (gray bars), and *mtrA*-deficient (*ΔmtrA*) mutant containing the vector pXM-$P_{NCgl1418}$-*gfp* was represented by red bars. All data represent mean values from three biological replicates including standard deviations.

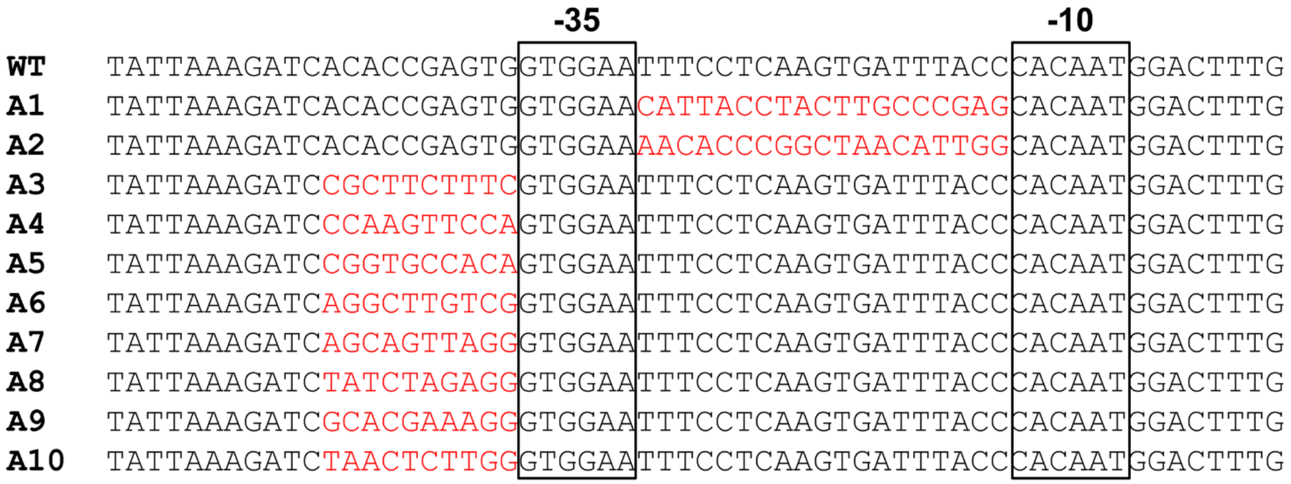

Figure S2. Sequence analysis of the isolated promoters. The boxes indicate the −10 and −35 regions of promoters. The WT promoter is $P_{NCgl1418}$.

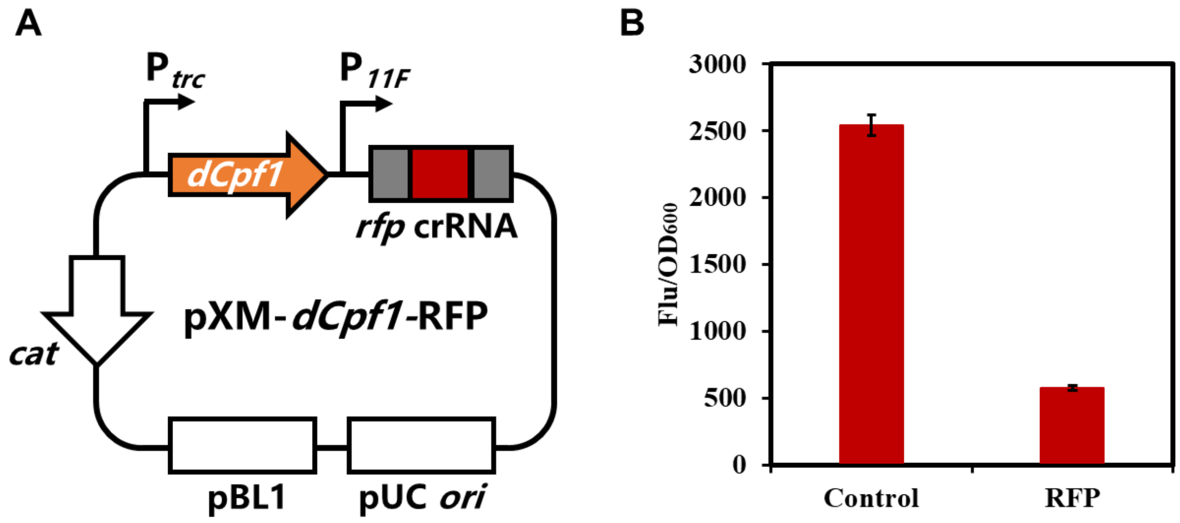

Figure S3. Development of an all-in-one plasmid for CRISPR-dCpf1-mediated gene repression in *C. glutamicum*. **(A)** Schematic diagram of the tool plasmid for *rfp* repression. **(B)** Fluorescence intensities of RFP controlled by the CRISPR-dCpf1 system with *rfp*-targeting crRNA. The strain expressing dCpf1 but no crRNA was used as the control. IPTG (1 mM) was used for inducing dCpf1 expression. Cell fluorescence intensity was normalized to cell density. All data represent mean values from three biological replicates including standard deviations.

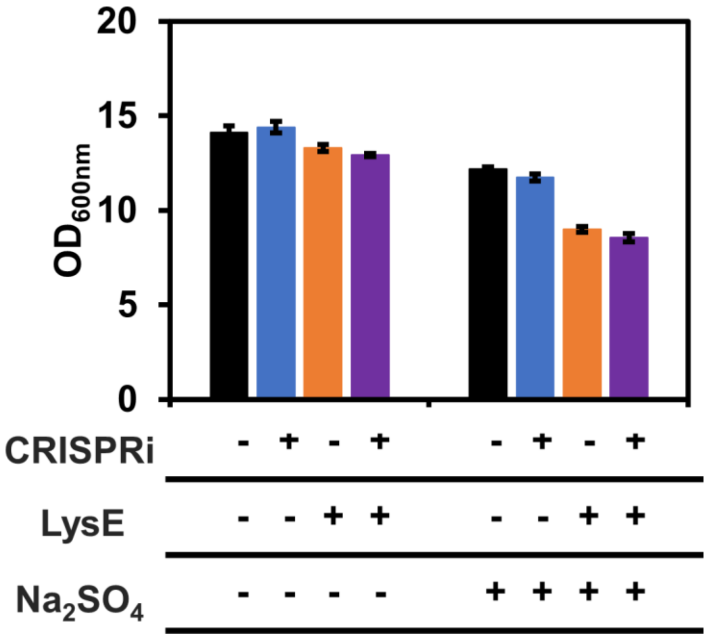

Figure S4. Effects of $P_{NCgl1418\text{-}A10}$ promoter-regulated *lysE* overexpression and/or multiple genes (*gltA*, *pgi*, *hom*, and *pck*) repression on cell densities. SCgL30 strains expressing LysE and/or dCpf1 with a crRNA array were cultivated in fermentation medium for 36 h. SCgL30 strains containing the empty vector pEC-XK99E and/or the plasmid pXM-$P_{NCgl1418}$-con without the crRNA array were used as controls. All data represent mean values from three biological replicates including standard deviations.

Table S1 Bacterial strains and plasmids used in this study

Strain or plasmid	Relevant characteristic[a]	Source
**Strain**		
E. coli		
Trans1-T1	Host for cloning	TransGen Biotech
Trans DB 3.1	Host for cloning of plasmid harboring *ccdB* gene	TransGen Biotech
C. glutamicum		
ATCC 13032	Wild-type strain	ATCC
ATCC 13032::*rfp*	ATCC 13032 derivative with chromosomal insertion of a *rfp* expression cassette	(Liu et al., 2017)
SCgL30	ATCC 13032 derivative with LysC^{T311I}	Lab stock
ATCC 13032 Δ*mtrA*	ATCC 13032 derivative with *mtrA* deleted	This study
SGcon	ATCC 13032 derivative harboring plasmid pXM-con-*gfp*	This study
SGabgT	ATCC 13032 derivative harboring plasmid pXM-P_{abgT}-*gfp*	This study
SGcsbD	ATCC 13032 derivative harboring plasmid pXM-P_{csbD}-*gfp*	This study
SGbetP	ATCC 13032 derivative harboring plasmid pXM-P_{betP}-*gfp*	This study
SG1418	ATCC 13032 derivative harboring plasmid pXM-$P_{NCgl1418}$-*gfp*	This study
SG1756	ATCC 13032 derivative harboring plasmid pXM-$P_{NCgl1756}$-*gfp*	This study
SG1838	ATCC 13032 derivative harboring plasmid pXM-$P_{NCgl1838}$-*gfp*	This study
SG2841	ATCC 13032 derivative harboring plasmid pXM-$P_{NCgl2841}$-*gfp*	This study
SGproP	ATCC 13032 derivative harboring plasmid pXM-P_{proP}-*gfp*	This study
SGtac	ATCC 13032 derivative harboring plasmid pXM-P_{tac}-*gfp*	This study
SGtuf	ATCC 13032 derivative harboring plasmid pXM-P_{tuf}-*gfp*	This study
SG203	ATCC 13032 derivative harboring plasmid pXM-$P_{NCgl1418-203}$-*gfp*	This study
SG145	ATCC 13032 derivative harboring plasmid pXM-$P_{NCgl1418-145}$-*gfp*	This study
SG94	ATCC 13032 derivative harboring plasmid pXM-$P_{NCgl1418-94}$-*gfp*	This study
SMcon	ATCC 13032 Δ*mtrA* derivative harboring plasmid pXM-con-*gfp*	This study
SM1418	ATCC 13032 Δ*mtrA* derivative harboring plasmid pXM-$P_{NCgl1418}$-*gfp*	This study
SRcon	ATCC 13032::*rfp* derivative harboring plasmid pXM-07	This study
SRRFP	ATCC 13032::*rfp* derivative harboring plasmid pXM-*dCpf1*-RFP	This study
SLDEcon1	SCgL30 derivative harboring plasmids pXM-$P_{NCgl1418}$-con and pEC-XK99E	This study
SLD1418	SCgL30 derivative harboring plasmids pXM-$P_{NCgl1418}$-*dCpf1*-4crRNA and pEC-XK99E	This study
SLE1418	SCgL30 derivative harboring plasmids pEC-$P_{NCgl1418}$-*lysE* and pXM-$P_{NCgl1418}$-con	This study
SLDE1418	SCgL30 derivative harboring plasmids pXM-$P_{NCgl1418}$-*dCpf1*-4crRNA and pEC-$P_{NCgl1418}$-*lysE*	This study

SLDEcon2	SCgL30 derivative harboring plasmids pXM-$P_{NCgl1418\text{-}A10}$-con and pEC-XK99E	This study
SLDA10	SCgL30 derivative harboring plasmids pXM-$P_{NCgl1418\text{-}A10}$-*dCpf1*-4crRNA and pEC-XK99E	This study
SLEA10	SCgL30 derivative harboring plasmids pEC-$P_{NCgl1418\text{-}A10}$-*lysE* and pXM-A10-con	This study
SLDEA10	SCgL30 derivative harboring plasmids pXM-$P_{NCgl1418\text{-}A10}$-*dCpf1*-4crRNA and pEC-$P_{NCgl1418\text{-}A10}$-*lysE*	This study
**Plasmid**		
pXMJ19	Expression vector of *E. coli* and *C. glutamicum*, IPTG-inducible promoter P_{tac}, Cm^R	(Jakoby et al., 1999)
pEC-XK99E	Expression vector of *E. coli* and *C. glutamicum*, IPTG-inducible promoter P_{trc}, Km^R	(Kirchner and Tauch, 2003)
pXM-*gfp*	pXMJ19 derivative carrying *gfp* gene	(Sun et al., 2019)
pEC-02	pEC-XK99E derivative, carrying the crRNA cassette with *ccdB* gene	(Li et al., 2020)
pXM-07	pXMJ19 derivative, carrying dCpf1 (E1006A, D917A) encoding gene	(Li et al., 2020)
pCas9	pXMJ19 derivative, carrying *cas9* gene, driven by IPTG-inducible promoter P_{tac}	(Liu et al., 2017)
pgRNA2	pEC-XK99E derivative carrying gRNA targeting *ldhA*, driven by constitutive promoter P_{11F}	(Liu et al., 2017)
pXM-con-*gfp*	pXM-*gfp* derivative with deletion of P_{tac}	This study
pXM-P_{abgT}-*gfp*	pXM-*gfp* derivative, P_{tac} replaced with P_{abgT}	This study
pXM-P_{csbD}-*gfp*	pXM-*gfp* derivative, P_{tac} replaced with P_{csbD}	This study
pXM-P_{betP}-*gfp*	pXM-*gfp* derivative, P_{tac} replaced with P_{betP}	This study
pXM-$P_{NCgl1418}$-*gfp*	pXM-*gfp* derivative, P_{tac} replaced with $P_{NCgl1418}$	This study
pXM-$P_{NCgl1756}$-*gfp*	pXM-*gfp* derivative, P_{tac} replaced with $P_{NCgl1756}$	This study
pXM-$P_{NCgl1838}$-*gfp*	pXM-*gfp* derivative, P_{tac} replaced with $P_{NCgl1838}$	This study
pXM-$P_{NCgl2841}$-*gfp*	pXM-*gfp* derivative, P_{tac} replaced with $P_{NCgl2841}$	This study
pXM-P_{proP}-*gfp*	pXM-*gfp* derivative, P_{tac} replaced with P_{proP}	This study
pXM-P_{tuf}-*gfp*	pXM-*gfp* derivative, P_{tac} replaced with P_{tuf}	This study
pXM-P_{tac}-*gfp*	pXM-*gfp* derivative, the original RBS replaced with the RBS of $P_{NCgl1418}$	This study
pXM-$P_{NCgl1418\text{-}203}$-*gfp*	pXM-$P_{NCgl1418}$-*gfp* derivative, $P_{NCgl1418}$ replaced with $P_{NCgl1418\text{-}203}$	This study
pXM-$P_{NCgl1418\text{-}145}$-*gfp*	pXM-$P_{NCgl1418}$-*gfp* derivative, $P_{NCgl1418}$ replaced with $P_{NCgl1418\text{-}145}$	This study
pXM-$P_{NCgl1418\text{-}94}$-*gfp*	pXM-$P_{NCgl1418}$-*gfp* derivative, $P_{NCgl1418}$ replaced with $P_{NCgl1418\text{-}94}$	This study
pEC-$P_{NCgl1418}$-*lysE*	pEC-XK99E derivative, carrying *lysE* gene, P_{trc} replaced with $P_{NCgl1418}$	This study
pEC-$P_{NCgl1418\text{-}A10}$-*lysE*	pEC-$P_{NCgl1418}$-*lysE* derivative, $P_{NCgl1418}$ replaced with $P_{NCgl1418\text{-}A10}$	This study

pXM-*dCpf1*	pXM-07 derivative, carrying the crRNA cassette with *ccdB* gene	This study
pXM-*dCpf1*-RFP	pXM-*dCpf1* derivative, carrying the *rfp* crRNA	This study
pXM-*dCpf1*-4crRNA	pXM-*dCpf1* derivative, carrying the crRNA array targeting *gltA*, *pgi*, *hom*, and *pck* genes	This study
pXM-$P_{NCgl1418}$-con	pXM-07 derivative, P_{tac} replaced with $P_{NCgl1418}$	This study
pXM-$P_{NCgl1418}$-*dCpf1*-4crRNA	pXM-*dCpf1*-4crRNA derivative, P_{tac} replaced with $P_{NCgl1418}$	This study
pXM-$P_{NCgl1418\text{-}A10}$-con	pXM-$P_{NCgl1418}$-con derivative, $P_{NCgl1418}$ replaced with $P_{NCgl1418\text{-}A10}$	This study
pXM-$P_{NCgl1418\text{-}A10}$-*dCpf1*-4crRNA	pXM-$P_{NCgl1418}$-*dCpf1* derivative, $P_{NCgl1418}$ replaced with $P_{NCgl1418\text{-}A10}$	This study
pgRNA-ΔmtrA	pgRNA2 derivative, for knock-out of *mtrA*	This study

[a]Km^R and Cm^R represent resistance to kanamycin and chloramphenicol, respectively.

Table S2 Primers used in this study

Primer	Sequence (5'-3')	Relevance
pGFP-F	ATGAGTAAAGGAGAAGAACTTTTCAC	pXM-con-gfp construction
pGFP-R	CCCGTCTCACTGGTGAAAAG	
abgT-F	TTCACCAGTGAGACGGGCGTGTCCAAGGTAGAGCAGTTG	pXM-P$_{abgT}$ -*gfp* construction
abgT-R	TCTTCTCCTTTACTCATTTCTCAGACCTTTGCAGTCATG	
csbD-F	TTCACCAGTGAGACGGGATCGTATTCGGAGCATTTGAGC	pXM-P$_{csbD}$ -*gfp* construction
csbD-R	TCTTCTCCTTTACTCATGTGGTGCTCATCTCCTACTTCTAG	
betP-F	TTCACCAGTGAGACGGGAGCGGTTGAAAGCGGTGTGC	pXM-P$_{betP}$ -*gfp* construction
betP-R	TCTTCTCCTTTACTCATTATCGGTTTCGGATTTGGGTCAG	
1418-F	TTCACCAGTGAGACGGGTAAAACTCGCGATGAAGTAG	pXM-P$_{NCgl1418}$-*gfp* construction
1418-R	TCTTCTCCTTTACTCATCATTGGCCTTTCTCGAATTGGG	
1756-F	TTCACCAGTGAGACGGGAGGGTTGCTAACGGTAAAGAATG	pXM-P$_{NCgl1756}$-*gfp* construction
1756-R	TCTTCTCCTTTACTCATCGGGATTCCTTTCTGTTTTGC	
1838-F	TTCACCAGTGAGACGGGCGTCTGCGCAAAGAACTCAAC	pXM-P$_{NCgl1838}$-*gfp* construction
1838-R	TCTTCTCCTTTACTCATCAGCTGGCCTTTCCATCGAC	
2841-F	TTCACCAGTGAGACGGGATTGGTGTTTGCGGGTTAGTTC	pXM-P$_{NCgl2841}$-*gfp* construction
2841-R	TCTTCTCCTTTACTCATGTGGTTCTCCTTTTCTTTCGGTG	
proP-F	TTCACCAGTGAGACGGGGCACCGAAAACAGTAACTTTCC	pXM-P$_{proP}$ -*gfp* construction
proP-R	TCTTCTCCTTTACTCATGAGTAAAACCTCTCGTCATATCTTATGAG	
tuf-F	CACCAGTGAGACGGGAGATCGTTTAGATCCGAAGGAAAA	pXM-P$_{tuf+1418RBS}$ -*gfp* construction
tuf-R	CTCGAATTGGGTATCAACGGACTTCGTGGTGGCTACGA	
pGFP-tuf-F	GTTGATACCCAATTCGAGAAAGG	
tac-F	CGAGAAAGGCCAATGATGAGTAAAGGAGAAGAACTTTTCACTG	pXM-P$_{tac+1418RBS}$ -*gfp* construction
tac-R	AATTGGGTATCAACAAGCTTAATTAATTCTGTTTCCTGTGTG	

1418-203-F	GACACCTGTGAGTTTCAAACTCC	pXM-P$_{NCgl1418\text{-}206bp}$-*gfp* construction
1418-145-F	TTTGTTTATGCAGGTGGCG	pXM-P$_{NCgl1418\text{-}148bp}$-*gfp* construction
1418-94-F	GTTTCTATGTATTAAAGATCACACCG	pXM-P$_{NCgl1418\text{-}97bp}$-*gfp* construction
pEC-F	TTTCACACAGGAAACAGACCATG	pEC-P$_{NCgl1418}$-*lysE* construction
pEC-R	AACGTAAATGCATGCCGCTTC	
lysE-F	ATGGTGATCATGGAAATCTTCATTAC	
lysE-R	GTCTGTTTCCTGTGTGAAACTAACCCATCAACATCAGTTTGATG	
1418-E-F	AGCGGCATGCATTTACGTTTAAAACTCGCGATGAAGTAG	
1418-E-R	AGATTTCCATGATCACCATCATTGGCCTTTCTCGAATTGGG	
A10-E-F	TAACTCTTGGGTGGAATTTCCTCAAGTGATTTAC	pEC-P$_{A10}$-*lysE* construction
A10-E-R	GATCTTTAATACATAGAAACAGATCTGTTACC	
pXM-07-F1	GAATATAAGGTCTCAGACACCAGAAGACAAGAATTTCTACTGTTGTAGATCCAGGCATCAAATAAAACG	pXM-*dCpf1* construction
pXM-07-R1	ACCGAAGATAGCTCATGTTATATCCCGCCGTTA	
pXM-07-F2	CATGAGCTATCTTCGGTATCGTCGTATCCCA	
pXM-07-R2	CATGAAGATGGTACGCGACTGGGCGTGGA	
pXM-07-F3	TCGCGTACCATCTTCATGGGAGAAAATAATAC	
pXM-07-R3	TGAATTACACTGTACCTGTTGCG	
ccdB-F	GTACAGTGTAATTCAGAATTTCTACTGTTGTAGATTTGTCTTCTGGTGTCTGAGACCACGCGTGGATCC	
ccdB-R	TGAGACCTTATATTCCCCAGAACATCAG	
RFP-F	AGATAAAGTTCGTATGGAAGGTTCCGTT	pXM-*dCpf1*-RFP construction
RFP-R	ATTCAACGGAACCTTCCATACGAACTTT	
array-F1	AGATAAAGGGATATCGTGGCTACTGATGAATTTCTACT	pXM-*dCpf1*-4crRNA construction
array-R1	CAACAGTAGAAATTCATCAGTAGCCACGATATCCCTTT	
array-F2	GTTGTAGATGCAAGACCTGACCGATCATTACTGAATTTC	
array-R2	AGTAGAAATTCAGTAATGATCGGTCAGGTCTTGCATCTA	
array-F3	TACTGTTGTAGATACCCCGGCAAGGGTCCCGGCTCAGAATTTCT	
array-R3	CAGTAGAAATTCTGAGCCGGGACCCTTGCCGGGGTATCTACAAC	
array-F4	ACTGTTGTAGATTGTTCGTTGATGGATCCCAGGCT	
array-R4	ATTCAGCCTGGGATCCATCAACGAACAATCTACAA	
pXM-D-F1	GTGTCAATTTATCAAGAATTTGTTAATAAA	

pXM-D-R1	AACCGTATTACCGCCTTTGAG	pXM-$P_{NCgl1418}$-con and pXM-$P_{NCgl1418}$-*dCpf1* construction
pXM-D-F2	CAAAGGCGGTAATACGGTTATC	
pXM-D-R2	CCCGTCTCACTGGTGAAAAG	
1418-D-F	CTTTTCACCAGTGAGACGGGTAAAACTCGCGATGAAGTAG	
1418-D-R	TTCTTGATAAATTGACACCATTGGCCTTTCTCGAATTGGG	
A10-D-F1	TAACTCTTGGGTGGAATTTCCTCAAGTGATTTAC	pXM-P_{A10}-con and pXM-P_{A10}-*dCpf1* construction
A10-D-R1	AACCGTATTACCGCCTTTGAG	
A10-D-F2	CAAAGGCGGTAATACGGTTATC	
A10-D-R2	AAATTCCACCCAAGAGTTAGATCTTTAATACATAGAAACAGATCTGTTACC	
gRNA-mtrA	GTGGTGCTCGGTTTGGAATCGTTTTAGAGCTAGAAATAGCAAG	pgRNA-Δ*mtrA* construction
pgRNA-1	CGCTTCCTTTAGCAGCCCTTG	
pgRNA-2	GTAGAAAGCCAGTCCGCAGAAACG	
pgRNA-3	GATTCCAAACCGAGCACCACTGAATTACACTGTACCTGTTGCGTC	
mtrA-del-up-F	GGGCTGCTAAAGGAAGCGACCCATCATTTGTCATGTCCAAC	
mtrA-del-up-R	GTCGGTGACAGCTACGGTGT	
mtrA-del-down-F	ACCGTAGCTGTCACCGACACACTCGACTGGTCAACGTTC	
mtrA-del-down-R	TGCGGACTGGCTTTCTACCGTGTGAGACATCCGATGTG	
P-Library-F1	NNNNNNNNNNNNNNNNNNNNCACAATGGACTTTGTTGATACCC	promoter library construction
P-Library-R1	TTCCACCACTCGGTGTGATC	
P-Library-F2	NNNNNNNNNNGTGGAATTTCCTCAAGTGATTTACC	
P-Library-R2	GATCTTTAATACATAGAAACAGATCTGTTACCC	

Table S3 crRNAs and gene expression elements used in this study

Gene element	Sequence (5'-3')
**crRNA**	
rfp	GAATTTCTACTGTTGTAGATaaagttcgtatggaaggttccgttGAATTTCTACTGTTGTAGAT
crRNA array targeting four genes	GAATTTCTACTGTTGTAGATaaagggatatcgtggctactgatGAATTTCTACTGTTGTAGATgcaagacctgaccgatcattactGAATTTCTACTGTTGTAGATaccccggcaagggtcccggctcaGAATTTCTACTGTTGTAGATtgttcgttgatggatcccaggctGAATTTCTACTGTTGTAGAT
**Promoter**	
P_{tac}	TGACAATTAATCATCGGCTCGTATAATGTGTGGAATTGTGAGCGGATAACAATTTCACACAGGAAACAGAATTAATTAAGCTTAAGAAGGAGATATACAT
P_{trc}	TTGACAATTAATCATCGGCTCGTATAATGTGTGGAATTGTGAGCGGATAACAATTTC
P_{11F}	TTTTCTCCACATAAGCTGGCAATGTTGCGACGCAACAGGTACAGTGTAATTCA
P_{abgT}	CGTGTCCAAGGTAGAGCAGTTGAAGGATTTGATGGCTGTGAAGGATGTGGAGCTGAGCGCTGAGCAGCTTGCACGTTTGGATAAGGTTTCGGAGCCTTTCGCTTAAGCTCTCCTCAAAAGTAAGTGATGCAAGTCATAACATTTGCATTACGGTTGCCTTATTTACCGATATTTATTCGGTGAGTTACTTAGTCTGGCTGGCATCGCCGGAAAACCGGACCGAGGTTTTCCGCATGACTGCAAAGGTCTGAGAA
P_{csbD}	ATCGTATTCGGAGCATTTGAGCCCCGAACCGGCGCCTGTGGATCTGTATTTGACGTGGTTCGAGACCCCGCGGTGTTGCACAAAGTGGAAGTCAGTGGAGGAATCCTCGAGCCTGAATGTGCTGCCTTGATGACCGAATTTTTCGAACTTCACAGGTAACGGATTTATATCAATTTCAGGGCGTGGCGAGCTTTTAGTGATTCACGCTCCTACGGTGGGTATCACAAATACCTCAACTAGAAGTAGGAGATGAGCACCAC
P_{betP}	AGCGGTTGAAAGCGGTGTGCGCAGACCTCGCGAAAATCCATGGCGTGAGCAAAAATGAACTCTACGATGCGGTTATTTCTGCCAGGGAAAATTAATCGTTATGTCATTGTGATGCTCCCGTGAACATAAACGGGACTTACTGGCTTTACTTAAGTAACAGCTATGTAAAAGACCAGGTCAGGTTCGGGTCGGTTTAGGTACGAAACCCATTTTTCGGTTTGCTTTCCAGGTTTCCCCAAGTAAAGGTGAGTTTTATGACTACATCTGACCCAAATCCGAAACCGATA
$P_{NCgl1418}$	TAAAACTCGCGATGAAGTAGAAAAACAACGCAACACTTAAGACACCTGTGAGTTTCAAACTCCCCATTATCGCCTTAGTCAGGCGGTAGTGGGGAGTTTTTGTTTATGCAGGTGGCGCGATTCTTAGATTTCATAAGGGTAACAGATCTGTTTCTATGTATTAAAGATCACACCGAGTGGTGGAATTTCCTCAAGTGATTTACCCACAATGGACTTTGTTGATACCCAATTCGAGAAAGGCCAATG

$P_{NCgl1756}$	AGGGTTGCTAACGGTAAAGAATGAGAGTTTCGCCACTCTAAAATCGCG<br>GTGAGCAGGGCGGTTGCGATGACTAAAACCACGAAAGCAAGAAGGAA<br>AACTGAATACATTTCCGGAAGGGGGATATTGTTTGACGTATTGATTTC<br>GTTAGCGACATTCACCTGTGGTTTACTCCCCTGCTTTTCGACAATGTAG<br>TGGTCATACGTTTCGATCATTTGTAACATTCTTATTACAAGACTGGAAA<br>TAAAGGTGAGTCAATCATACAGTCAGATGTGGTCATCGGCAAAACAG<br>AAAGGAATCCCG
$P_{NCgl1838}$	CGTCTGCGCAAAGAACTCAACACCATCGTGTCTATCACCTCTGGACGC<br>ACCGGCCGCCCACACGGCGCAATCCACACCGAAGCACGTAAACACTG<br>CGGTGGCCCACCAACGGCACTTTGTAGCGCCGAACAACTCCGGGCACG<br>CATTGACTACCTGCGAAAGTGGTAACACATAACATTTTTGTTGCAATA<br>TCCATACCCTCAACATCTGCTCAAGGTGTTGAGGGTATCCTTGTCTGGG<br>CATAAAAATACATAGCTTTGTGCAGCATTTATTGAACCACGTCGATGG<br>AAAGGCCAGCTG
$P_{NCgl2841}$	ATTGGTGTTTGCGGGTTAGTTCGGGCCATTCGAAAGGGAGAAACCAAG<br>GGCAGCCAGACAGACGTGCCAAGAATCTGGATTTCCGCCAGGTTTTGG<br>CACGCCCGTCTGGTTTAGGCAATGAGATACCGAACACACGTGCCAAAA<br>GTTCGGCTTTTTCGCCGATCTTGTCACGCCTGCCTGGTTTGTCTTGTAA<br>AGAGTGATTTCATGGCCGAGACTCCTAAAAGTTTGACCTCACAGGATT<br>GCTTCTAAGGGCCTCTCCAATCTCCACTGAGGTACTTAATCCTTCCGGG<br>GAATTCGGGCGCTTAAATCGAGAAATTAGGCCATCACCTTTTAATAAC<br>AATACAATGAATAATTGGAATAGGTCGACACCTTTGGAGCGGAGCCG<br>GTTAAAATTGGCAGCATTCACCGAAAGAAAAGGAGAACCAC
P_{proP}	GCACCGAAAACAGTAACTTTCCCAAGAAAAATATAAGAAAACTTCCC<br>CACACAGGCCGTGAAGAGCCTGAATTTATTGATTTTTCAGACAGATCT<br>GGAAATGTGACCAATTTGTAACCCACCCCCGCTCACCTGCATGAGTGT<br>GGGGTCTTTTTGCATTCTTCCAGCTCCCAGACTTGAAAACGATCTGACT<br>TTTCACCCCGAACCTTACTAAGGTCGATTCATGTTGAAAAGAGAGGTG<br>GTGTTTTCACTTCCCTTTTATAGGCAAAGCTTTAAGGAGTCTTACAGGA<br>AGAAGTTAACACCGCCCAGGGGTGCGTTGGATGATGATCATCTACAAA<br>CAAACATTCCGTTATGCACTCATAAGATATGACGAGAGGTTTTACTC
P_{tuf}	AGATCGTTTAGATCCGAAGGAAAACGTCGAAAAGCAATTTGCTTTTCG<br>ACGCCCCACCCCGCGCGTTTTAGCGTGTCAGTAGGCGCGTAGGGTAAG<br>TGGGGTAGCGGCTTGTTAGATATCTTGAAATCGGCTTTCAACAGCATT<br>GATTTCGATGTATTTAGCTGGCCGTTACCCTGCGAATGTCCACAGGGT<br>AGCTGGTAGTTTGAAAATCAACGCCGTTGCCCTTAGGATTCAGTAACT<br>GGCACATTTTGTAATGCGCTAGATCTGTGTGCTCAGTCTTCCAGGCTGC<br>TTATCACAGTGAAAGCAAAACCAATTCGTGGCTGCGAAAGTCGTAGCC<br>ACCACGAAGTCCGTTGATACCCAATTCGAGAAAGGCCAATG
P_{tac}	TGACAATTAATCATCGGCTCGTATAATGTGTGGAATTGTGAGCGGATA<br>ACAATTTCACACAGGAAACAGAATTAATTAAGCTTGTTGATACCCAAT<br>TCGAGAAAGGCCAATG
$P_{NCgl1418\text{-}203}$	GACACCTGTGAGTTTCAAACTCCCCATTATCGCCTTAGTCAGGCGGTA<br>GTGGGGAGTTTTTGTTTATGCAGGTGGCGCGATTCTTAGATTTCATAAG<br>GGTAACAGATCTGTTTCTATGTATTAAAGATCACACCGAGTGGTGGAA<br>TTTCCTCAAGTGATTTACCCACAATGGACTTTGTTGATACCCAATTCGA<br>GAAAGGCCAATG

P$_{NCgl1418-145}$	TTTGTTTATGCAGGTGGCGCGATTCTTAGATTTCATAAGGGTAACAGATCTGTTTCTATGTATTAAAGATCACACCGAGTGGTGGAATTTCCTCAAGTGATTTACCCACAATGGACTTTGTTGATACCCAATTCGAGAAAGGCCAATG
P$_{NCgl1418-94}$	GTTTCTATGTATTAAAGATCACACCGAGTGGTGGAATTTCCTCAAGTGATTTACCCACAATGGACTTTGTTGATACCCAATTCGAGAAAGGCCAATG
P$_{NCgl1418-A10}$	TAAAACTCGCGATGAAGTAGAAAAACAACGCAACACTTAAGACACCTGTGAGTTTCAAACTCCCCATTATCGCCTTAGTCAGGCGGTAGTGGGGAGTTTTTGTTTATGCAGGTGGCGCGATTCTTAGATTTCATAAGGGTAACAGATCTGTTTCTATGTATTAAAGATCTAACTCTTGGGTGGAATTTCCTCAAGTGATTTACCCACAATGGACTTTGTTGATACCCAATTCGAGAAAGGCCAATG

[a]Direct repeat and spacer sequences of crRNAs are indicated as capital and lowercase letters, respectively.

References

Jakoby, M., Ngouoto-Nkili, C.E., and Burkovski, A. (1999). Construction and application of new *Corynebacterium glutamicum* vectors. *Biotechnol. Tech.* 13, 437–441. doi: Doi 10.1023/A:1008968419217

Kirchner, O., and Tauch, A. (2003). Tools for genetic engineering in the amino acid-producing bacterium *Corynebacterium glutamicum*. *J. Biotechnol.* 104, 287–299. doi: 10.1016/S0168-1656(03)00148-2

Li, M., Chen, J., Wang, Y., Liu, J., Huang, J., Chen, N., et al. (2020). Efficient multiplex gene repression by CRISPR-dCpf1 in *Corynebacterium glutamicum*. *Front. Bioeng. Biotechnol.* 8, 357. doi: 10.3389/fbioe.2020.00357

Liu, J., Wang, Y., Lu, Y., Zheng, P., Sun, J., and Ma, Y. (2017). Development of a CRISPR/Cas9 genome editing toolbox for *Corynebacterium glutamicum*. *Microb. Cell. Fact.* 16, 205. doi: 10.1186/s12934-017-0815-5

Sun, D.H., Chen, J.Z., Wang, Y., Li, M.Y., Rao, D.M., Guo, Y.M., et al. (2019). Metabolic engineering of *Corynebacterium glutamicum* by synthetic small regulatory RNAs. *J. Ind. Microbiol. Biotechnol.* 46, 203–208. doi: 10.1007/s10295-018-02128-4
